# Nursing students’ clinical practice education experience during the COVID-19 pandemic: a qualitative study

**DOI:** 10.1186/s12912-024-01730-5

**Published:** 2024-01-23

**Authors:** Soo Jin Kwon, Yoonjung Kim, Yeunhee Kwak

**Affiliations:** 1 Department of Nursing, Ansan University, Ansan, Republic of Korea; 2 Faculty of Red Cross College of Nursing, Chung-Ang University, 156-756 Seoul, Republic of Korea

**Keywords:** Anxiety, Attitude, Clinical practice, Education, Nursing students, Qualitative research

## Abstract

**Background:**

Nursing education, including some elements of clinical practice, has largely been conducted online during the coronavirus disease 2019 (COVID-19) pandemic. Numerous studies have examined the experiences of nursing students in academia during the pandemic. However, research on nursing students’ clinical practice experiences is limited. This study aimed to analyze nursing students’ clinical practice experiences during the COVID-19 pandemic.

**Methods:**

This study used a qualitative research design and performed thematic analysis. Participants comprised 13 nursing university students with clinical practice experience at a hospital during the COVID-19 pandemic. Interviews were conducted either online or face-to-face. Data were collected during June‒July 2021.

**Results:**

Four themes and eight subthemes were generated through thematic analysis. Theme 1 was “Nursing students’ anxiety and strict adherence to quarantine practices,” with the subthemes of “Fear of infection” and “Protecting themselves.” Theme 2 was “Nursing students’ belief that their organization is protecting them,” with the subthemes of “Strict quarantine rules at hospitals and universities” and “The nursing students regretted the limited scope of practice but felt safe.” Theme 3 was “Learning through valuable practice,” with the subthemes of “Recognizing the importance of practice” and “Realizing the benefits of practice.” Theme 4 was “A sense of duty as a prospective nurse,” with the subthemes of “Accepting a sense of duty as a nurse” and “Establishing the expanded role of a nurse.”

**Conclusions:**

The nursing students recognized the importance of nursing practice during the pandemic and worked harder. A better understanding of the experiences of nursing university students who completed their clinical practice during the COVID-19 pandemic can help nursing professors and managers more effectively train students during times of high stress.

**Supplementary Information:**

The online version contains supplementary material available at 10.1186/s12912-024-01730-5.

## Background

At the end of 2019, the coronavirus disease 2019 (COVID-19) began to spread worldwide [1]. As of 2021, South Korea had reported 749,979 confirmed cases and 6,588 deaths; the social, economic, and educational chaos continues to this day [2]. Although medical professionals were prioritized for vaccination, many of them still died from COVID-19 [2]. Clinical practice is an essential part of nursing schools’ curriculums to train competent nurses; however, as an increasing number of clinical training institutions either suspended or discontinued practice considering patient and student safety and the government’s quarantine guidelines, clinical practice was temporarily suspended or transformed into non-face-to-face online practice [3]. This situation was very flexible, with on-site clinical practice being suspended and resumed based on the government’s social distancing guidelines, the situation of the clinical training institution, and the policy of the institution.

Clinical practice is the primary component of nursing education that enables students to engage in professional roles and acquire the behaviors, attitudes, and values that are essential to the nursing profession [4, 5]. However, nursing students experienced anxiety, stress, and emotional problems because of clinical practice during the pandemic [6, 7].

The world is currently facing a mental health crisis following the radical public health interventions that were implemented during the pandemic and the ongoing health-related effects of COVID-19 [8]. According to the Korean National Mental Health Survey, 48% of South Koreans reported experiencing anxiety and depression because of the COVID-19 pandemic [9]. Therefore, understanding nursing students’ clinical practice experiences in situations where COVID-19 could threaten their mental health is critical.

A Belgian analysis of nursing students’ experiences during clinical placement indicated their general satisfaction with the support provided by their universities; however, despite such positive efforts, students reported experiencing confusion, including practical worries, reduced learning opportunities, and fundamental doubts about their choice to become nurses [10]. Spanish final-year nursing students who were hired to help the national health system’s COVID-19 response reported being proud of their contribution to their country [11].

Most South Korean studies of nursing students’ practical experience have only explored online nursing practice [3, 12, 13]. Therefore, in-depth research from various perspectives is necessary to understand nursing students’ experiences of clinical practice at hospitals during the COVID-19 crisis. The current study’s findings could help prepare and guide nursing students for clinical practice, as well as inform universities, in the future.

This study analyzed the experiences of nursing students practicing at a hospital during the COVID-19 pandemic. We intend to use these findings as basic data for analyzing and improving nursing education and guidance.

### Aim

This study analyzed nursing students’ clinical practice experiences during the COVID-19 pandemic. The relevant data represent a foundation for providing education and safety guidance.

## Methods

### Design

This was a descriptive qualitative study that used Braun and Clarke’s thematic analysis method [15]. The data were obtained through individual in-depth interviews with nursing students. When preparing the manuscript, the Consolidated Criteria for Reporting Qualitative Research (COREQ checklist) was followed.

### Participants

We used convenience sampling to recruit participants. The study participants were nursing university students aged 19 years or older who had gained some clinical practice experience at a hospital at least twice during the COVID-19 pandemic. A notice inviting students to participate was posted on the selected university’s bulletin board, and the students who volunteered were recruited. Recruitment was stopped when data saturation was reached. All 13 participants (10 women and 3 men) were unmarried. Moreover, six were third-year university students and seven were in their fourth year. Their ages ranged from 22 to 27 years (Table 1). No participants withdrew from the study during the data collection and analysis period.

**Table 1:** Participants’ demographic data (*N* = 13)

Profile variables		N
Sex	Male Female	3 10
Age (years)	22 23 24 25 27	5 4 2 1 1
Grade	Junior (3rd year) Senior (4th year)	6 7
Marital status	Single	13

### Procedure

Data were collected during June–July 2021. To ensure participant safety during the pandemic, interviews were conducted online through video calls using Zoom^™^. Zoom is easy to use, cost-effective, and has several data management features and security options; the current study, therefore, adopted it based on a previous study that demonstrated its viability for qualitative data collection [14]. As the participants could only be observed through the Zoom screen, they were instructed to inform the interviewer before performing an action, such as thinking; for example, if they looked away from the screen to think, they informed the interviewer that they were thinking. One participant who requested a face-to-face interview was interviewed in the first author’s laboratory at a convenient time while all parties were wearing masks and observing the necessary COVID-19 safety protocols. COVID-19 safety precautions were maintained at all times during the study.

The interviews, each lasting 60–90 min, were recorded. The interviews were conducted by the first author. The interview questionnaire comprised semi-structured questions (Supplementary file 1); the key question was “What is your experience of clinical practice in a hospital in the context of COVID-19?” The researcher, who encouraged participants to describe their experiences as elaborately as possible, recorded all observed non-verbal responses and other notes in a field notebook at personal discretion. The data were transcribed based on the recorded interviews.

### Data analysis

The collected data were analyzed using Braun and Clarke’s [15] thematic analysis method to reveal the meaning of any common themes that emerged from the data. The thematic analysis method comprises six steps. Step 1 involves becoming familiar with the data. Repeated readings of the transcribed data allowed the researchers to identify meaningful content regarding the participants’ experiences during their COVID-19-related hospital practice. Step 2 involves generating initial codes; 230 meaningful statements were extracted and coded. Step 3 involves identifying a theme. The code was then compared with the potential theme, all data related to the potential theme were collected, and 45 potential themes were derived. Step 4 involves examining the themes. The researchers checked whether the extracted themes were consistent with the overall data. Finally, four themes and eight subthemes were identified. Step 5 involves naming each theme based on its meaning. Step 6 involves preparing a report. Accordingly, the researchers finally checked the relevant contents and described the analysis results.

### Rigor

To increase the rigor of the qualitative thematic analysis, throughout the study and until the conclusion of the findings, we continuously reviewed and analyzed the data to verify its reliability [16]. Finally, we asked participants to check the results to ensure the credibility of the findings.

## Results

The study identified four themes and eight subthemes (Table 2). The participants followed the quarantine rules conscientiously, as they were anxious, believed hospitals to be safe, recognized the importance of precautionary practices, and displayed a sense of duty as prospective nurses.

**Table 2:** Analysis of nursing students’ clinical practice education experience

Themes	Subthemes	Statements
Nursing students’ anxiety and strict adherence to quarantine rules	Fear of infection	*What would I do if I got infected with COVID-19? However, I think I was a little more worried that I might cause harm to others and not that I would get sick. (Participant 6)*
	Protecting themselves	*I still wash my hands a lot, and I think that I was washing them more than I thought I would when I attended practice. (Participant 6)*
Nursing students’ belief that their organization is protecting them	Strict quarantine rules at hospitals and universities	*When I went to the hospital, I saw that the hospital staff were controlling the infection very thoroughly and systematically, so I became less worried while practicing. (Participant 11)*
	The nursing students regretted the limited scope of practice but felt safe	*I think that COVID-19 limited students’ practice experience. We could only observe because of worries about infection. (Participant 2)*
Learning through valuable practice	Recognizing the importance of practice	*It is very difficult to get a job without any practical experience. We were lucky and thankful that we were able to practice as well. (Participant 5)*
	Realizing the benefits of practice	*Whenever I saw something new or something I had learned in theory, I immediately moved to experience it, and it seemed to become more memorable and unforgettable. (Participant 9)*
A sense of duty as a prospective nurse	Accepting a sense of duty as a nurse	*Frankly speaking, I am afraid of the infection, but if I become a nurse, I cannot choose the patient, so I think it is fate to some extent. (Participant 3)*
	Establishing the expanded role of a nurse	*I often think that I feel that nursing is a more difficult job than I had thought. I thought that is a job that requires a greater sense of duty than I had thought. (Participant 13)*

### Theme 1. Nursing students’ anxiety and strict adherence to quarantine rules

Owing to their anxiety about contracting COVID-19 during their practice and infecting and harming those around them (family members, colleagues, patients, and fellow nurses at the hospital), the students tried to diligently adhere to the quarantine rules.

#### Subtheme 1. Fear of infection

The participants showed greater anxiety about being infected with COVID-19 in public transportation services (e.g., buses and subways) while traveling to their training sites rather than being infected at the hospital. Although they were worried about becoming ill themselves, they showed greater anxiety about putting their family members and the people at the hospital at risk of infection, as well as any ensuing damage to the university.

*What would I do if I got infected with COVID-19? However, I think I was a little more worried that I might cause harm to others and not that I would get sick. After my parents were diagnosed with COVID-19, I thought that this was not a simple thing. If I got infected, it would harm the training center, the patients, and the school. (Participant 6)*

#### Subtheme 2. Protecting themselves

The participants strived to minimize any risk of infection and sought to protect themselves by using their knowledge regarding droplet dissemination, which they had acquired through university classes and the government’s quarantine guidelines. They emphasized the importance of wearing a proper mask and sanitizing and washing their hands; furthermore, they prioritized hand disinfection when leaving the hospital premises. They limited their offline interpersonal interactions and indicated that they had no choice but to interact with friends using online platforms such as Zoom.

*I still wash my hands a lot, and I think that I was washing them more than I thought I would when I attended practice. When I attended practice, my fingertips and skin were often scratched here and there. I now wash my hands a lot—even when I come home. When someone goes out and comes home, I tell them to wash their hands before entering the house. (Participant 6)*

### Theme 2. Nursing students’ belief that their organization is protecting them

The participants were aware of the expertise and efforts of their universities and hospitals regarding the prevention of COVID-19 infection and transmission. They believed that hospitals were safe and stated that, although they regretted that some aspects of the practice were limited, they were able to engage in practice with relatively less anxiety.

#### Subtheme 1. Strict quarantine rules at hospitals and universities

Some participants stated that they felt safer at hospitals compared to daily living environments (e.g., restaurants, cafes, bars, and public transportation). Based on their expertise, the students thought that the hospitals had appropriate management mechanisms (e.g., access control, COVID-19 testing for inpatients and caregivers, limits on patient visits, and isolation of high-infection-risk patients). The universities also educated students about reducing infection risk by avoiding multi-use facilities and minimizing outings for two weeks before practice; furthermore, they managed the activities of students in accordance with strict guidelines (e.g., requiring students to write a self-checklist related to COVID-19 and testing them for COVID-19 before practice). As described above, the participants reported that they were able to engage in practice with trust, thanks to the strict quarantine rules implemented by the universities and hospitals.

*People around me were very worried about whether I would get infected by a patient after going to the hospital or whether I would get infected by the nurses I worked with, and I was also very worried at first. However, when I went to the hospital, I saw that the hospital staff were controlling the infection very thoroughly and systematically, so I became less worried while practicing. (Participant 11)*

#### Subtheme 2. The nursing students regretted the limited scope of practice but felt safe

The participants were instructed in advance to not approach high-infection-risk patients and were provided with protective equipment to avoid transmission. Although they regretted that their scope of practice was limited owing to COVID-19, they stated that they could practice with confidence because they felt that the hospital had implemented the restrictions to protect them.

*I think that COVID-19 limited students’ practice experience. I think that we could have experienced various additional skills; however, there was no choice, and we could only observe because of worries about infection. (Participant 2)*

### Theme 3. Learning through valuable practice

The participants were forced to engage only in online practice in the past year because they had to comply with social distancing regulations as part of COVID-19 preventive measures; they recognized the seriousness and importance of clinical practice. When practice resumed, the participants engaged more diligently during the training period, as they were more aware of its benefits.

#### Subtheme 1. Recognizing the importance of practice

Initially, although the participants feared the COVID-19 situation, they expected it to improve with time. However, when the situation did not change, they realized the importance of their previous daily life and practice and understood that they needed to develop their capabilities through their own efforts while accepting the current situation. Moreover, they realized the importance of practice for nursing students and acknowledged it as a necessary part of their experience during their university studies. As practice could be suspended or changed depending on the situation, they hoped that the COVID-19 situation would not worsen during the periods when practice was available; thus, they participated enthusiastically in clinical practice because they perceived that it could be interrupted at any time.

*Last year, I thought that the school would stop practice for the safety of the students. This year, fourth-year students must get a job; however, it is very difficult to get a job without any practical experience. We were lucky and thankful that we were able to practice as well. (Participant 5)*

#### Subtheme 2. Realizing the benefits of practice

The participants stated that they found practice useful because it allowed them to apply what they had learned in theory and experience it first-hand. Thus, they felt that they could expand and supplement their knowledge through practice; furthermore, as nursing students, they realized its benefits.

*When I was in the first semester of my third year, I went to practice three times. Whenever I saw something new or something I had learned in theory, I immediately moved to experience it, and it seemed to become more memorable and unforgettable. (Participant 9)*

### Theme 4. A sense of duty as a prospective nurse

The participants experienced pride in their identities as nurses; this feeling was strengthened by the changes in their awareness regarding nurses’ roles in the COVID-19 pandemic, which had been fostered by the media. Furthermore, when they observed nurses contributing to the COVID-19 pandemic efforts by playing their roles as medical personnel, the students began to naturally accept their responsibilities as nurses at hospitals and toward society. Moreover, they expressed a sense of duty as prospective nurses while establishing their expanded nursing roles.

#### Subtheme 1. Accepting a sense of duty as a nurse

Though, as nurses, the participants experienced several difficulties while working, primarily owing to COVID-19 quarantine rules and infection control regulations, they accepted these rules and regulations rather than viewing them as a burden.

*Frankly speaking, I am afraid of the infection, but if I become a nurse, I cannot choose the patient, so I think it is fate to some extent. (Participant 3)*

*Ever since I engaged in practice, I think that, even if I become a nurse in the future, it will be natural for me to do the work of a quarantine nurse, and I think I will do the same. (Participant 11)*

#### Subtheme 2. Establishing the expanded role of a nurse

The participants felt proud to be nurses, as the number of news broadcasts, programs, and advertisements regarding nurses increased during the COVID-19 pandemic, and social awareness about nurses changed positively. Additionally, the media coverage and their own practice increased participants’ awareness regarding nurses’ roles and abilities in a global crisis such as the COVID-19 pandemic. The students’ participation in practice allowed them to concretize an expanded role of nurses within hospitals and society.

*I often think that I feel that nursing is a more difficult job than I had thought. Originally, I knew it was hard, but observing how they work more—through the media and in person—has made me think that it is a job that requires a greater sense of duty than I had thought. I thought more seriously about becoming a nurse, and I wanted to become a tireless nurse. (Participant 13)*

*I saw nurses in the media wearing protective gear and struggling with COVID-19, and I often thought that, when I gained some skills, if an infectious disease such as COVID-19 were to return, I would go where my help was required. Seeing such people, I felt that they had a strong professional spirit as nurses, and because of them, my awareness about nurses greatly increased, which was wonderful. (Participant 11)*

## Discussion

This study explored the clinical practice experiences of nursing students who were working during the COVID-19 pandemic.

The first theme was that the nursing students tried to follow the quarantine rules conscientiously to protect themselves and others from the fear and anxiety of infection risk. Studies of nursing students who engaged in clinical practice during the COVID-19 pandemic have shown that such nurses tended to experience much anxiety and feared the risk of infection, and experienced a high amount of stress concerning the new virus [17, 18]. Another study also reported similar findings regarding nursing students’ fear; primarily, they “thought that they could become a confirmed case” owing to the spread of COVID-19 and “followed personal infection prevention guidelines” to avoid infection [19]. In addition to fear of infection, nurses also expressed anxiety over becoming a source of contamination and further spreading the infection [20]. Such anxieties concerning exposure to infection and the fear of transmission may weaken nursing activities, and safe nursing is, therefore, essential when responding to infectious diseases [21]. Therefore, education regarding infection control and pandemics should be disseminated to nursing students in a systematic, repetitive, and diverse manner.

During the pandemic, students’ psychological stress increased due to fear and anxiety about infection risk, which reduced their capacity to develop professional identity [18]. Nursing institutions and nurse educators can improve psychological well-being by reducing nursing students’ stress and strengthening protective factors such as resilience and self-efficacy [22]. Thus, governments should provide psychological support services to address these problems [9]. Efforts are needed to provide a sense of stability through systematic emotional support.

The second theme was that most nursing students reported that although they were anxious, they were still able to engage in practice because they were confident in the safety of hospitals and the protection provided to them by the hospital and university systems.

Nursing students confirmed their belief in the organization that they were protected and their belief in social support. This trust may be related to their belief that their organization would respond adequately and provide sufficient support during a pandemic [23]. The pandemic situation may have led to reduced learning opportunities and even raised fundamental doubts about their choice of becoming nurses [10]. Therefore, a clinical practice program can strengthen the capabilities of nursing students, and it is necessary to emphasize the awareness that they are sufficiently protected during clinical practice.

The third theme was learning through valuable practice. In this study, the selected nursing students believed that practice would aid their employment as nurses in the future, and they were therefore eager to engage in practice even in difficult situations. They stated that they learned a great deal by being more engaged during the training period because the experience was valuable. Although the students were confused at the beginning of the pandemic, their clinical practice experience helped them discover themselves, gain a stronger identity as a nurse, and acknowledge their inner maturity for managing problems by adopting an introspective approach [19]. For nursing students, clinical practice, thus, provides opportunities to learn practical skills, form an identity as a nurse, and mature internally. When there are limitations in clinical practice, it is necessary to maximize the effectiveness of practical education through research related to confidence and competency as new nurses, and to maintain the quality of continuous education by improving clinical practice education programs.

The last theme was that, during difficult situations, clinical practice served as an opportunity to expand knowledge about infectious diseases and awareness of the role of nurses, thereby providing an opportunity to grow as a prospective health guardian. This is consistent with previous findings that nursing students’ image of nurses and professionalism as nurses significantly improved after their first clinical practice in the COVID-19 situation compared with before practice [2]. Thus, while nursing students experienced physical and mental weakness because of fear of infection and stress of clinical practice, they simultaneously laid the foundation for becoming professional nurses by using various strategies as trainees and maintaining the learning environment [19, 24]. As they observed nurses perform their roles as medical personnel during the pandemic, the students naturally began to accept their responsibilities as prospective nurses and feel a sense of duty while establishing the expanded role of a nurse.

Effective clinical teaching and the development of nursing students’ competence in clinical practice can only occur in supportive clinical learning environments with fully engaged students [4]. This study showed that nursing students experienced the positive and negative aspects of nursing during clinical practice and that it helped them develop an awareness and identity as professional nurses. Subsequently, it is necessary to build a professional clinical practice field and create an institutional plan for establishing nursing competence, which, in turn, will allow students to experience high-quality clinical practice even during a pandemic.

The results of this study can contribute to the development of nursing practice by influencing the formation of nursing students’ positive career views and their growth as professional nurses. Furthermore, this study has educational significance in that nursing students witnessed nurses working on the frontlines in the pandemic, became aware of the meaning and importance of the role of nurses, and redefined the nursing profession.

This study had limitations. First, the participants were recruited from only one South Korean university. Accordingly, the generalization of the study’s results to different COVID-19 situations, countries, regions, and cultures should be considered with some caution. Second, during the period of participants’ engagement in practice, the medical staff had received the COVID-19 vaccine but had not (for various reasons) vaccinated all students. Accordingly, it should be noted that more widespread dissemination of the vaccination to the students could have changed their experiences and thoughts. Third, there are differences in the role of nurses and the nursing work environment depending on the size of the clinical practice hospital or the hospital situation, which may result in differences in the clinical practice experience of nursing students.

Based on the results of this study, university and hospital officials need to develop strategies for the clinical practice system and practice content so that clinical practice can be safely conducted even if there is another pandemic in the future. Furthermore, empirical research is needed to explore the clinical adaptation experience of new nurses who graduated after COVID-19 and identify the impact and difference between face-to-face and non-face-to-face clinical practice experience on nurses’ clinical performance ability.

## Conclusions

This study analyzed the experiences nursing students gained through clinical practice during the COVID-19 pandemic from their perspective. Although nursing students experienced considerable anxiety and stress due to COVID-19, they recognized the importance of clinical practice and established their roles as nurses. By recognizing the importance of nursing amidst a global disaster, the participants developed a sense of pride in nursing and a sense of duty as prospective nurses. Therefore, universities and hospitals should strictly follow the quarantine rules and promote awareness about infections among students to enable their performance of clinical practice in a safe environment. Additionally, universities and other related stakeholders must provide them with support to enhance their competence as prospective nurses. It is necessary to develop strategies for efficient clinical practice through continuous analysis of various aspects of clinical practice types.

### Electronic supplementary material

Below is the link to the electronic supplementary material.

Supplementary Material 1

## Data Availability

The full interviews´ data are not publicly available due to privacy or ethical restrictions. However data is available upon reasonable request from the corresponding author.
