# Supplementary material for: Nursing students’ clinical practice education experience during the COVID-19 pandemic: a qualitative study
Source: BMC Nurs. 2024 Jan 23;23:63. doi: 10.1186/s12912-024-01730-5 (PMC10807203; doi:10.1186/s12912-024-01730-5)
Supplement: Supplementary file 1 — Supplementary Material 1 [file 12912_2024_1730_MOESM1_ESM.docx]

**Interview questions**

The questionnaire below was used during the interview.

Hello.

From now on, I will be interviewing you about how you felt while practicing at a hospital during the coronavirus pandemic.

1. What did you think when you heard that you would be doing hospital training during the coronavirus situation? What did it feel like?

2. So, what thoughts or feelings did you have while practicing at the hospital?

Please tell us in detail what you felt or thought during the practice.

3. The number of confirmed coronavirus cases increased during practice. What did you think at that time?

4. How did you feel about the quarantine measures at the hospital and teachers during your practicum?

5. How did you prepare while practicing during the coronavirus pandemic?

6. How has the coronavirus pandemic affected you?

7. How did your thoughts change before and after the practicum?

If you have anything else you would like to say as we conclude the interview, please feel free to do so. Thank you.
